# Supplementary material for: Cohesin Mutations Induce Chromatin Conformation Perturbation of the H19/IGF2 Imprinted Region and Gene Expression Dysregulation in Cornelia de Lange Syndrome Cell Lines
Source: Biomolecules. 2021 Nov 2;11(11):1622. doi: 10.3390/biom11111622 (PMC8615450; doi:10.3390/biom11111622)
Supplement: Supplementary file 1 [file biomolecules-11-01622-s001.zip › Figure S1.pdf]

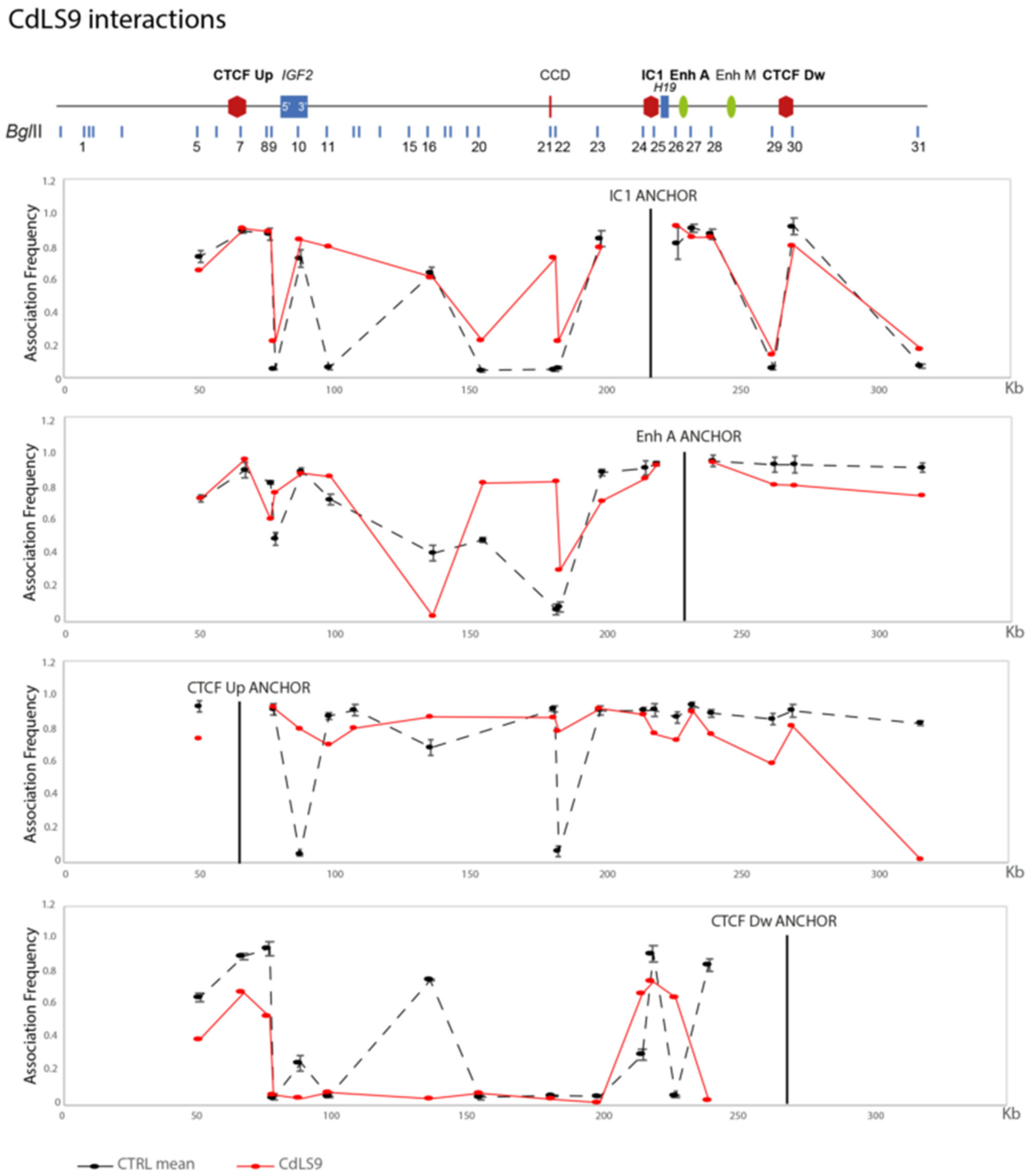

Figure S1. Abnormalities in chromatin architecture at the IC1 *locus* of the CdLS9 cell line (*NIPBL* mutation). IC1 *locus* looping profiles for the indicated anchors in controls (dotted black) and CdLS9 (red) cell lines. BglII restriction sites are indicated above. Each point in the profile is the mean ± standard deviation of two independent 3C experiments and indicates the association frequency between the anchor and the fragment on the left of the corresponding BglII restriction site.
