# Supplementary material for: Cohesin Mutations Induce Chromatin Conformation Perturbation of the H19/IGF2 Imprinted Region and Gene Expression Dysregulation in Cornelia de Lange Syndrome Cell Lines
Source: Biomolecules. 2021 Nov 2;11(11):1622. doi: 10.3390/biom11111622 (PMC8615450; doi:10.3390/biom11111622)
Supplement: Supplementary file 1 [file biomolecules-11-01622-s001.zip › Figure S2.pdf]

a

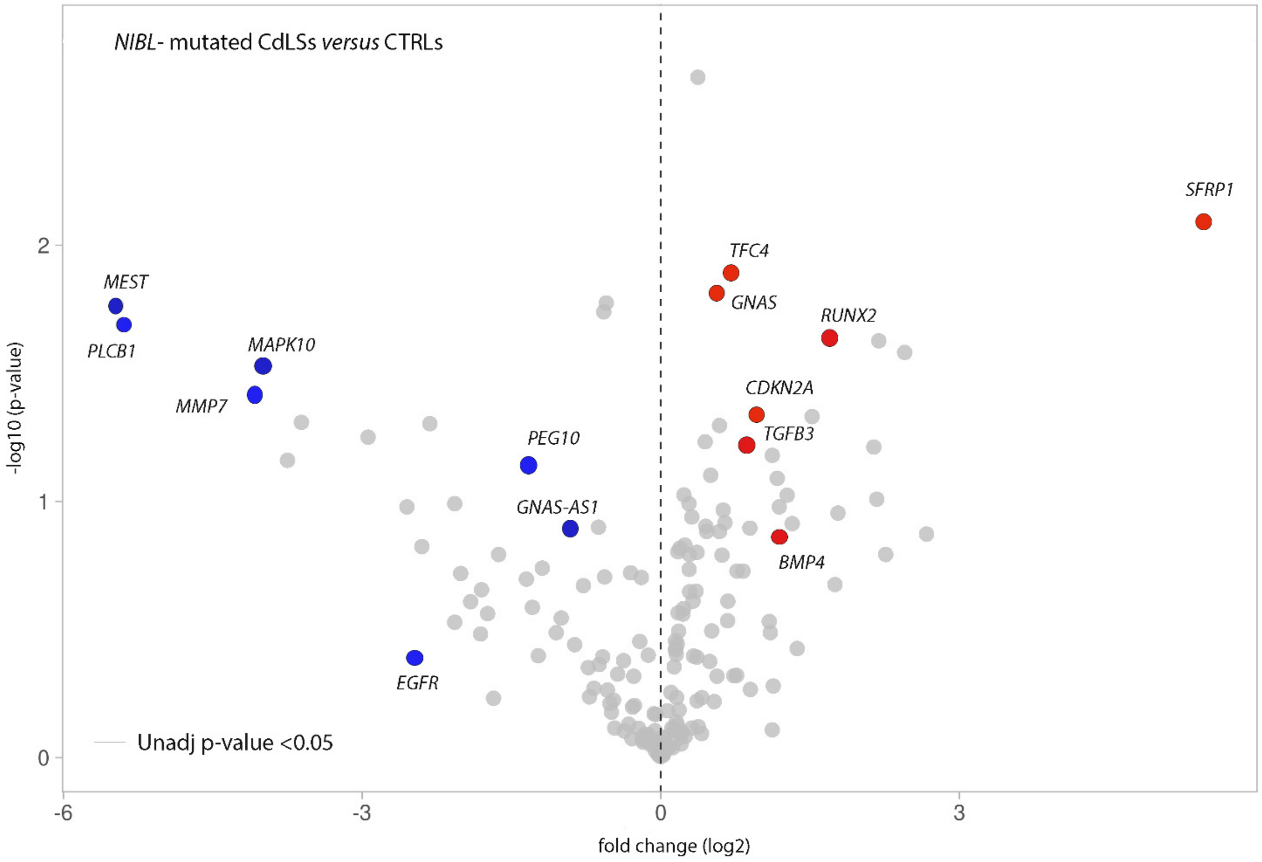
NIBL- mutated CdLSs *versus* CTRLs
SFRP1
TFC4
GNAS
RUNX2
CDKN2A
TGFB3
BMP4
MEST
PLCB1
MAPK10
MMP7
PEG10
GNAS-AS1
EGFR
Unadj p-value <0.05
-log10 (p-value)
2
1
0
-6
-3
0
3
fold change (log2)

GENE	Accession	Log2 fold change	P-value	BH p-value	Pathway annotation
CUL1	NM_003592.2	0.373	0.00221	0.375	KEGG WNT Annotation
SFRP1	NM_003012.3	5.45	0.00807	0.402	Canonical Wnt Pathway, KEGG WNT Annotation, WNT Signaling Negative Regulation
TCF4	NM_003199.1	0.712	0.0128	0.402	Transcription Factors
GNAS	NM_080425.1	0.573	0.0153	0.402	
BIRC5	NM_001168.2	-0.548	0.0168	0.402	Cell Cycle
MEST	NM_177525.1	-5.47	0.0172	0.402	
TBL1XR1	NM_024665.4	-0.573	0.0182	0.402	KEGG WNT Annotation
PLCB1	NM_182734.1	-5.4	0.0204	0.402	KEGG WNT Annotation
RUNX2	NM_004348.3	1.69	0.0231	0.402	Development & Differentiation, Transcription Factors
TLE1	NM_005077.3	2.19	0.0236	0.402	WNT Signaling Negative Regulation
AXIN2	NM_004655.3	2.45	0.0262	0.405	Canonical Wnt Pathway, KEGG WNT Annotation, WNT Signaling Negative Regulation, WNT Signaling Target Genes
MAPK10	NM_002753.2	-4	0.0295	0.418	KEGG WNT Annotation
MMP7	NM_002423.3	-4.08	0.0382	0.474	Calcium Binding and Signaling, KEGG WNT Annotation, Proteolysis, WNT Signaling Target Genes
CDKN2A	NM_000077.3	0.96	0.0455	0.474	Cell Cycle, Development & Differentiation, Transcription Factors
IRS1	NM_005544.2	1.52	0.0466	0.474	Migration
FZD5	NM_003468.2	-3.61	0.0491	0.474	Canonical Wnt Pathway, KEGG WNT Annotation
PTGS2	NM_000963.1	-2.32	0.0497	0.474	Calcium Binding and Signaling, Cell Cycle

b

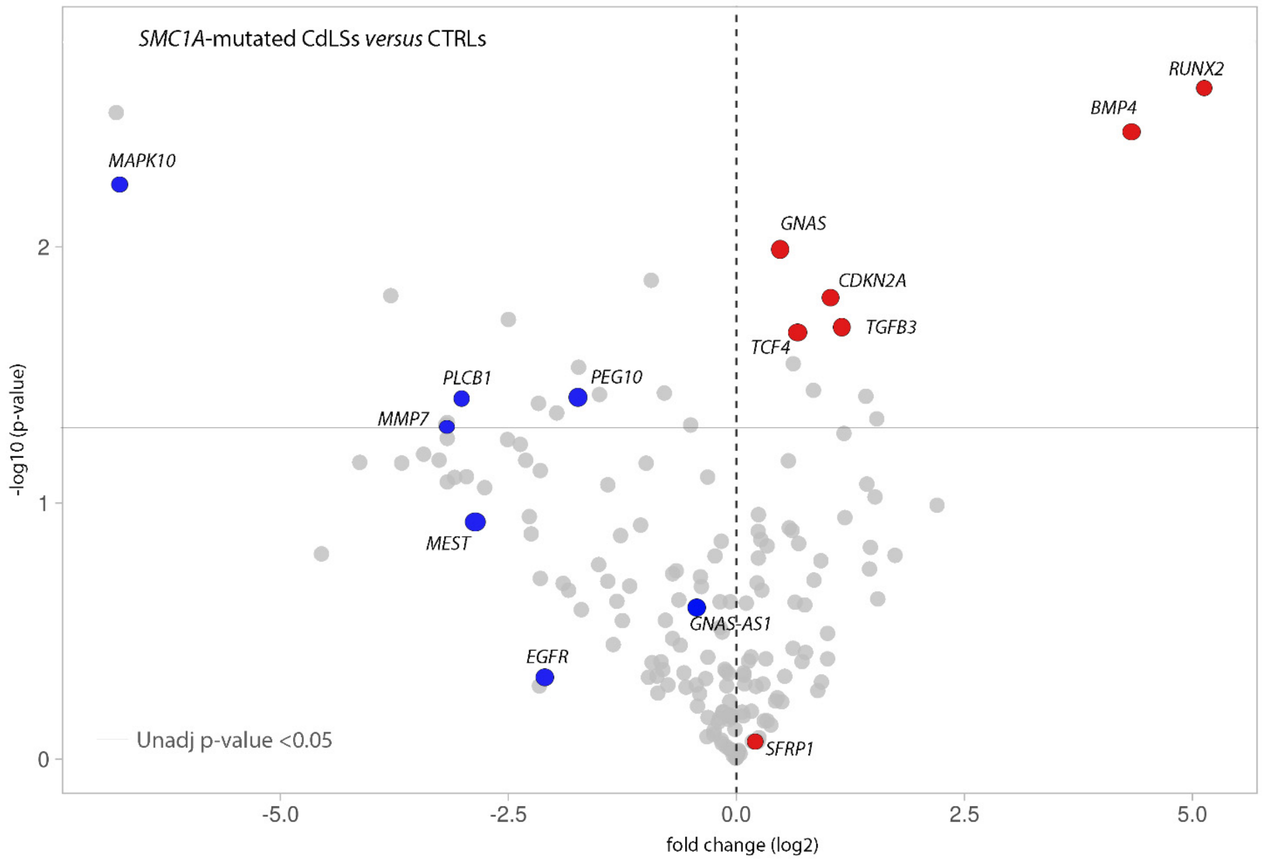

SMC1A-mutated CdLSs *versus* CTRLs
RUNX2
BMP4
MAPK10
GNAS
CDKN2A
TGFB3
TCF4
PLCB1
PEG10
MMP7
MEST
GNAS-AS1
EGFR
SFRP1
Unadj p-value <0.05
-log10 (p-value)
2
1
0
-5.0
-2.5
0.0
2.5
5.0
fold change (log2)

GENE	Accession	Log2 fold change	P-value	BH p-value	Pathway annotation
RUNX2	NM_004348.3	5.12	0.0024	0.209	Development & Differentiation, Transcription Factors
PTGS2	NM_000963.1	-6.8	0.00299	0.209	Calcium Binding and Signaling, Cell Cycle
BMP4	NM_001202.3	4.33	0.00356	0.209	Development & Differentiation
MAPK10	NM_002753.2	-6.75	0.00571	0.251	KEGG WNT Annotation
GNAS	NM_080425.1	0.476	0.0102	0.342	
PLAUR	NM_001005376.1	-0.934	0.0135	0.342	Proteolysis
KREMEN1	NM_001039570.1	-3.79	0.0155	0.342	WNT Signaling Negative Regulation
CDKN2A	NM_000077.3	1.03	0.0158	0.342	Cell Cycle, Development & Differentiation, Transcription Factors
CXCR4	NM_003467.2	-2.5	0.0192	0.342	EMTMetastasis
TGFB3	NM_003239.2	1.16	0.0205	0.342	Development & Differentiation
TCF4	NM_003199.1	0.683	0.0218	0.342	Transcription Factors
NLK	NM_016231.2	0.624	0.0286	0.342	KEGG WNT Annotation, WNT Signaling Negative Regulation
H19	NR_002196.1	-1.73	0.0295	0.342	
FZD3	NM_017412.2	0.845	0.0363	0.342	Canonical Wnt Pathway, KEGG WNT Annotation
MAPK8	NM_002750.2	-0.79	0.0372	0.342	KEGG WNT Annotation, Planar Cell Polarity (PCP)
TIMP1	NM_003254.2	-1.5	0.0377	0.342	EMTMetastasis
FRAT1	NM_005479.3	1.42	0.0383	0.342	Canonical Wnt Pathway, KEGG WNT Annotation
PEG10	NM_001040152.1	-1.73	0.0384	0.342	
PLCB1	NM_182734.1	-3.02	0.0392	0.342	KEGG WNT Annotation
FZD6	NM_003506.2	-2.17	0.0408	0.342	Canonical Wnt Pathway, KEGG WNT Annotation
SOX9	NM_000346.2	-1.97	0.0445	0.342	Adhesion, Development & Differentiation, Transcription Factors
WNT8B	NM_003393.2	1.54	0.0469	0.342	KEGG WNT Annotation
MMP7	NM_002423.3	-3.17	0.0485	0.342	Calcium Binding and Signaling, KEGG WNT Annotation, Proteolysis, WNT Signaling Target Genes
PPARD	NM_006238.3	-0.502	0.0496	0.342	Development & Differentiation, KEGG WNT Annotation, Migration, Transcription Factors, WNT Signaling Target Genes
GDNF	NM_000514.2	-3.19	0.0496	0.342	Development & Differentiation, Migration

c

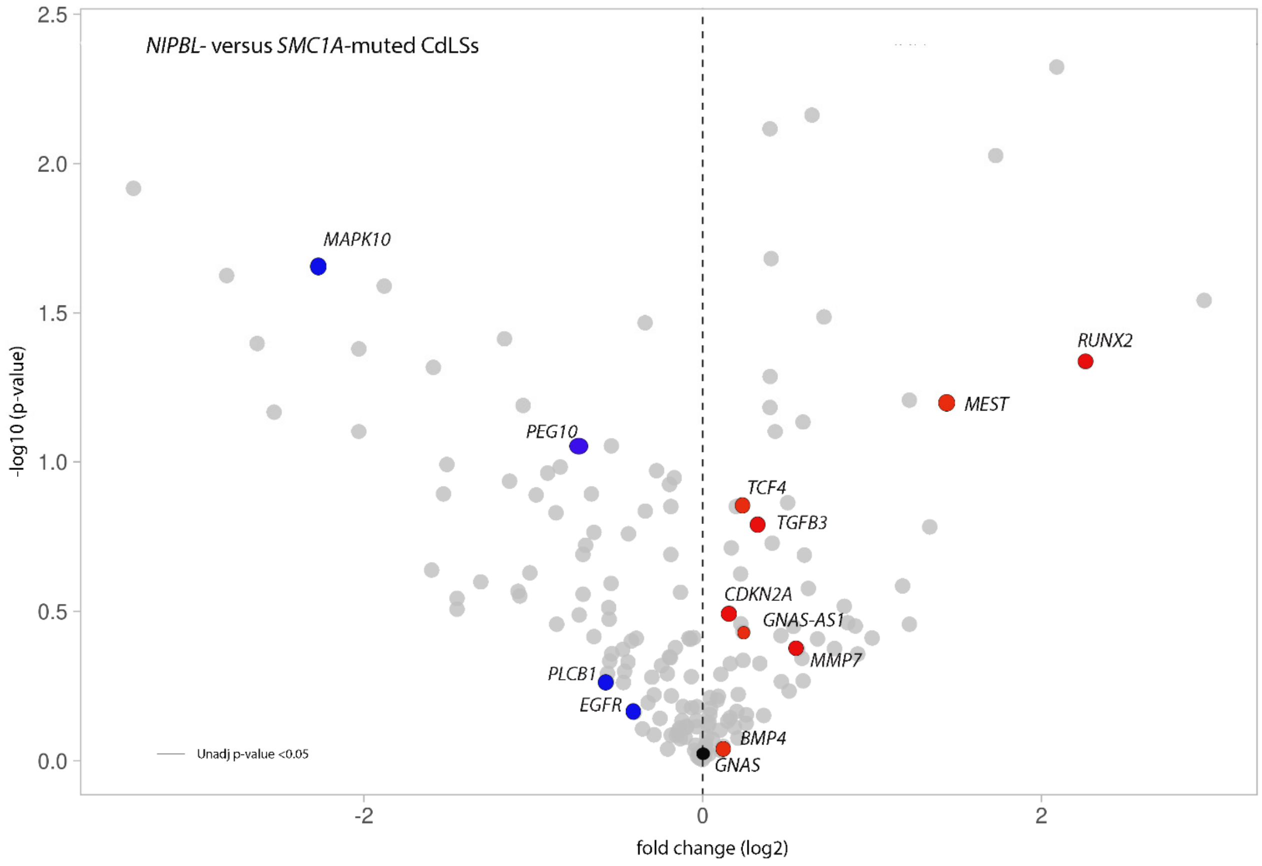

GENE	Accession	Log2 fold change	P-value	BH p-value	Pathway annotation
FZD5	NM_003468.2	2.09	0.00475	0.416	Canonical Wnt Pathway, KEGG WNT Annotation
NFATC1	NM_172389.1	0.645	0.00688	0.416	Calcium Binding and Signaling, KEGG WNT Annotation
TBL1XR1	NM_024665.4	0.397	0.00765	0.416	KEGG WNT Annotation
PRKCA	NM_002737.2	1.73	0.0094	0.416	KEGG WNT Annotation
NRCAM	NM_005010.4	-3.36	0.0121	0.427	Adhesion, Development & Differentiation, Migration
EP300	NM_001429.2	0.404	0.0208	0.493	Canonical Wnt Pathway, KEGG WNT Annotation
MAPK10	NM_002753.2	-2.27	0.0221	0.493	KEGG WNT Annotation
T	NM_003181.2	-2.81	0.0237	0.493	Development & Differentiation, Transcription Factors
FGF7	NM_002009.3	-1.88	0.0257	0.493	Migration
WNT7B	NM_058238.1	2.96	0.0287	0.493	Calcium Binding and Signaling, Canonical Wnt Pathway, KEGG WNT Annotation
POU5F1	NM_002701.4	0.716	0.0326	0.493	Development & Differentiation, Transcription Factors
FAM50B	NM_012135.1	-0.34	0.0341	0.493	
FZD6	NM_003506.2	-1.17	0.0386	0.493	Canonical Wnt Pathway, KEGG WNT Annotation
IGF1R	NM_000875.4	-2.63	0.04	0.493	

WNT7A	NM_004625.3	-2.03	0.0417	0.493	Calcium Binding and Signaling, Canonical Wnt Pathway, KEGG WNT Annotation
RUNX2	NM_004348.3	2.26	0.0462	0.501	Development & Differentiation, Transcription Factors
CDH11	NM_001797.2	-1.59	0.0481	0.501	EMTMetastasis

Figure S2. Three different expression profiles of CdLS samples split by *NIPBL* and *SMC1A* variations evaluated by nCounter Nanostring technology. A) *NIPBL*-mutated CdLSs versus CTRLs. B) *SMC1A*-mutated CdLSs versus CTRLs. C) *NIPBL*-mutated versus *SMC1A* -mutated CdLSs. Volcano plots (Top) and panels (Bottom) of significant DEGs. Upregulated and downregulated genes shared with the main cohort (CdLSs compared to CTRLs) are highlighted by red and blue dots, respectively. Unadjusted p-value <0.05 is indicated by horizontal line.
