# Supplementary material for: Cohesin Mutations Induce Chromatin Conformation Perturbation of the H19/IGF2 Imprinted Region and Gene Expression Dysregulation in Cornelia de Lange Syndrome Cell Lines
Source: Biomolecules. 2021 Nov 2;11(11):1622. doi: 10.3390/biom11111622 (PMC8615450; doi:10.3390/biom11111622)
Supplement: Supplementary file 1 [file biomolecules-11-01622-s001.zip › Table S1.pdf]

Table S1: Customized panel of imprinted and imprinted related genes analyzed by NCounter Nanostring approach

GENE	Accession	Position	Target Sequence	Tot Isoforms	Isoforms Hit By Probe
CDKN1C	NM_000076.2	1606-1705	TGTCCAGGCGTGGACCGCTCTGCCACGCACTAGCTCGGTTATTGGTTATGCC AAAGGCACTCTCCATCTCCCACATCTGGTTATTGACAAGTGTAACTTT	5	5
DIRAS3	NM_004675.2	951-1050	CTGAGAAGCTGCTTGACAAGTGCATAATCATGTGAGCCCTGGGCCTTAAGA GCCAGCTCTTCCTATCCTGTAGCGTGTAGAAAACGTGGACTCATTTCAC	1	1
DLK1	NM_003836.4	1329-1428	AGATTCTTGGAGTTCCGCAGAGCTTACTATACGCGGTCTGTCCTAATCTTTG TGGTGTTCGCTATCTCTTGTGTCAAATCTGGTGAACGCTACGCTTACA	2	2
FAM50B	NM_012135.1	1273-1372	TTCTGAGTATTTTAGTGTTGCCACCTGGATTTGCTGCATTGCTCTGCTGAGC TGTATTGAAACCATGACTGGGCCCACTGTCAGACAGAAATTAGAATAG	3	2
GNAS	NM_080425.1	1911-2010	CGAGAACCAGTTCAGAGTGGACTACATCCTGAGTGTGATGAACGTGCCTGA CTTTGACTTCCCTCCCGAATTCTATGAGCATGCCAAGGCTCTGTGGGAG	29	24
GNAS-AS1	NR_002785.2	1027-1126	ACGTCCGCTGGGAACTGGCTGCTCTGCAGGTTTCTGTATCACATTTTCTGCA CATGTCCATTAGAATTGGAGATGGGGCGTATCTAGTGTTGAATAAAGG	1	1
H19	NR_002196.1	1593-1692	GGCCTTTGAATCCGGACACAAAACCCTCTAGCTTGGAAATGAATATGCTGC ACTTTACAACCACTGCACTACCTGACTCAGGAATCGGCTCTGGAAGGTG	3	3
IGF1R	NM_000875.4	4581-4680	TCTCCTGAGTCCCTCAAGGATGGAGTCTTCACCACTTACTCGGACGTCTGGT CCTTCGGGGTCGTCCTCTGGGAGATCGCCACACTGGCCGAGCAGCCCT	9	9
IGF2	NM_001127598.1	2418-2517	ACACACACTCATGCGCAGCACATACATGAACACAGCTCACAGCACACAAA CACGCAGCACACACGTTGCACACGCAAGCACCCACCTGCACACACACATG	7	3
IGF2R	NM_000876.1	2606-2705	TGCCGGGCTGCAACCGATATGCATCGGCTTGCCAGATGAAGTATGAAAAAG ATCAGGGCTCCTTCACTGAAGTGGTTTCCATCAGTAACTTGGGAATGGC	1	1
INSR	NM_000208.2	526-625	CACCCGGGGTTCTGTCCGCATCGAGAAGAACAATGAGCTCTGTTACTTGGC CACTATCGACTGGTCCCGTATCCTGGATTCCGTGGAGGATAATTACATC	4	4

KCNQ1	NM_181798.1	836-935	CAGACGTGGGTCGGGAAGACCATCGCCTCCTGCTTCTCTGTCTTTGCCATCT CCTTCTTTGCGCTCCCAGCGGGGATTCTTGGCTCGGGGTTTGCCCTGA	2	2
KCNQ1OT1	NR_002728.2	31876-31975	GCCAGACATCAAATTGAGTGAGGGACATTGGACACAAAGGTGTATGAGTT GCATCATTCCCACCGCATAAAGTTCAAATGTGGGCAAAGCTGGTCCTTGC	1	1
MEG3	NR_002766.2	692-791	ATCTTCCTATCCTTTTCTGGGGGAATGGGGTCGATGAGAGCAACCTCCTAG GGTTGTTGTGAGAATTAAATGAGATAAAAGAGGCCTCAGGCAGGATCTG	15	15
MEST	NM_177525.1	646-745	CTCTGTCTGTCAAATGGAGGTATCTTTCCTGAGACTCACCGTCCACTCCTTC TCCAAAAGCTACTCAAAGATGGAGGTGTGCTGTCACCCATCCTCACAC	10	10
PEG10	NM_001040152.1	5001-5100	TTTGCCACCACTGCAAGCAAAAGTCTGGAGAAGTTCACCAACGACAAGAAC GATTAGGGAAAATATGCTGCTGTGGGTTAACAACTCAGAAAGTCCCTGA	6	6
PLAGL1	NM_006718.3	1873-1972	TCAGTTTGTTTGAGGACTTGCCTCTGCAAGAGCCTCAGTCACCTCAAAAGCT CAACCCAGGTTTTGATCTGGCTAAGGGAAATGCTGGTAAAGTAAACCT	27	27
