# Supplementary material for: Cohesin Mutations Induce Chromatin Conformation Perturbation of the H19/IGF2 Imprinted Region and Gene Expression Dysregulation in Cornelia de Lange Syndrome Cell Lines
Source: Biomolecules. 2021 Nov 2;11(11):1622. doi: 10.3390/biom11111622 (PMC8615450; doi:10.3390/biom11111622)
Supplement: Supplementary file 1 [file biomolecules-11-01622-s001.zip › Table S2.pdf]

Table S2a. Panel of imprinted genes and imprinted related genes analyzed by NCounter Nanostring approach

GENE	Accession	Log2 fold change	*p*-value	BH *p*-value	Annotation
GNAS	NM_080425.1	0.494	0.00166	0.0887	Imprinted gene
MEST	NM_177525.1	-3.65	0.0109	0.13	Imprinted gene
PEG10	NM_001040152.1	-1.56	0.0127	0.131	Imprinted gene
GNAS-AS1	NR_002785.2:1026	-0.709	0.0578	0.256	Imprinted lncRNA
KCNQ1OT1	NR_002728.2:31875	-0.535	0.0643	0.277	Imprinted lncRNA
INSR	NM_000208.2:525	-0.895	0.169	0.436	Imprinted related gene
IGF2R	NM_000876.1:2605	0.324	0.261	0.563	Imprinted related gene
FAM50B	NM_012135.1:1272	0.197	0.289	0.594	Imprinted gene
PLAGL1	NM_006718.3:1872	0.222	0.414	0.698	Imprinted gene
Genes below the background level					
IGF2	NM_001127598.1:2417	<20 counts			Imprinted Gene, Development & Differentiation, Cell Cycle
CDKN1C	NM_000076.2:1605	<20 counts			Imprinted gene
H19	NR_002196.1:1592	<20 counts			Imprinted lncRNA
MEG3	NR_002766.2:691	<20 counts			Imprinted lncRNA
IGF1R	NM_000875.4:4580	<20 counts			Imprinted related gene
DLK1	NM_003836.4:1328	<20 counts			Imprinted gene
KCNQ1	NM_181798.1:835	<20 counts			Imprinted gene
DIRAS3	NM_004675.2:950	<20 counts			Imprinted gene

Table S2b. Panel of genes of the WNT pathway analyzed by NCounter Nanostring approach

**GENE**	**Accession**	**Log2 fold change**	***p*-value**	**BH *p*-value**	**Pathway annotation**
RUNX2	NM_004348.3	4.25	0.00151	0.0887	Development & Differentiation, Transcription Factors
CDKN2A	NM_000077.3	0.971	0.00187	0.0887	Cell Cycle, Development & Differentiation, Transcription Factors
MAPK10	NM_002753.2	-4.76	0.002	0.0887	KEGG WNT Annotation
EGFR	NM_201282.1	-6.04	0.0028	0.0919	Adhesion, Calcium Binding and Signaling, Cell Cycle, Development & Differentiation, Migration
SFRP1	NM_003012.3	4.41	0.00411	0.0919	Canonical Wnt Pathway, KEGG WNT Annotation, WNT Signaling Negative Regulation
TCF4	NM_003199.1	0.661	0.00481	0.0919	Transcription Factors
BMP4	NM_001202.3	3.43	0.00524	0.0919	Development & Differentiation
PLCB1	NM_182734.1	-3.82	0.00524	0.0919	KEGG WNT Annotation
MMP7	NM_002423.3	-3.53	0.00556	0.0919	Calcium Binding and Signaling, KEGG WNT Annotation, Proteolysis, WNT Signaling Target Genes
TGFB3	NM_003239.2	0.978	0.00571	0.0919	Development & Differentiation
PTGS2	NM_000963.1	-3.26	0.00834	0.123	Calcium Binding and Signaling, Cell Cycle
TLE1	NM_005077.3	1.8	0.0106	0.13	WNT Signaling Negative Regulation
FZD5	NM_003468.2	-3.03	0.011	0.13	Canonical Wnt Pathway, KEGG WNT Annotation
CXCL12	NM_000609.5	-3.11	0.0132	0.131	EMT Metastasis
GDNF	NM_000514.2	-2.85	0.0133	0.131	Development & Differentiation, Migration
IRS1	NM_005544.2	1.3	0.016	0.149	Migration
FRAT1	NM_005479.3	1.25	0.0185	0.164	Canonical Wnt Pathway, KEGG WNT Annotation
FZD3	NM_017412.2	0.699	0.0211	0.173	Canonical Wnt Pathway, KEGG WNT Annotation
PPP3CC	NM_005605.4	0.552	0.0216	0.173	KEGG WNT Annotation

SNAI2	NM_003068.3	-2.73	0.0249	0.191	EMT Metastasis
KREMEN1	NM_001039570.1	-2.52	0.0268	0.191	WNT Signaling Negative Regulation
FZD10	NM_007197.2	-2.43	0.027	0.191	KEGG WNT Annotation
LEF1	NM_016269.3	1.83	0.0334	0.22	Canonical Wnt Pathway, KEGG WNT Annotation, Transcription Factors
NLK	NM_016231.2	0.519	0.0342	0.22	KEGG WNT Annotation, WNT Signaling Negative Regulation
SOX2	NM_003106.2	-1.74	0.0348	0.22	Cell Cycle, Development & Differentiation, Transcription Factors
PPP3CA	NM_000944.4	1.06	0.0401	0.237	KEGG WNT Annotation
CXCR4	NM_003467.2	-1.63	0.0418	0.237	EMT Metastasis
BIRC5	NM_001168.2	-0.491	0.0427	0.237	Cell Cycle
WNT10B	NM_003394.2	-1.26	0.0442	0.237	KEGG WNT Annotation
SERPINE1	NM_001165413.1	-2.36	0.0442	0.237	EMT Metastasis
TCF7L1	NM_031283.1	0.931	0.0477	0.24	Canonical Wnt Pathway, KEGG WNT Annotation, Transcription Factors
FZD8	NM_031866.1	-2.35	0.0479	0.24	Canonical Wnt Pathway, KEGG WNT Annotation
SMAD2	NM_005901.5	0.165	0.0488	0.24	EMTMetastasis, KEGG WNT Annotation
JUN	NM_002228.3	1.17	0.0541	0.255	KEGG WNT Annotation, WNT Signaling Target Genes
PRKACA	NM_002730.3	-0.24	0.0552	0.255	KEGG WNT Annotation
PPARD	NM_006238.3	-0.384	0.0562	0.255	Development & Differentiation, KEGG WNT Annotation, Migration, Transcription Factors, WNT Signaling Target Genes
RUVBL1	NM_003707.2	-0.226	0.0707	0.291	Canonical Wnt Pathway, KEGG WNT Annotation
TBL1XR1	NM_024665.4	-0.327	0.0764	0.307	KEGG WNT Annotation
PPAP2B	NM_003713.3	-2.69	0.0804	0.316	Migration
COL1A2	NM_000089.3	1.89	0.0859	0.328	EMT Metastasis
NKD1	NM_033119.3	0.658	0.0872	0.328	Canonical Wnt Pathway, KEGG WNT Annotation, Planar Cell Polarity (PCP), WNT Signaling Negative Regulation
FOXN1	NM_003593.2	-1.89	0.0917	0.338	Development & Differentiation
CTBP1	NM_001328.2	0.163	0.101	0.365	Canonical Wnt Pathway, KEGG WNT Annotation, WNT Signaling Negative

					Regulation
DIXDC1	NM_00103795.3	0.784	0.112	0.387	Canonical Wnt Pathway
DAB2	NM_001343.2	-1.62	0.132	0.436	Development & Differentiation, WNT Signaling Target Genes
DVL2	NM_004422.2	-0.182	0.137	0.436	Canonical Wnt Pathway, KEGG WNT Annotation, Planar Cell Polarity (PCP)
CUL1	NM_003592.2	0.147	0.138	0.436	KEGG WNT Annotation
WNT8B	NM_003393.2	1.15	0.139	0.436	KEGG WNT Annotation
RAC2	NM_002872.3	0.262	0.139	0.436	KEGG WNT Annotation
WNT2B	NM_024494.1	0.807	0.141	0.436	Calcium Binding and Signaling, Canonical Wnt Pathway, KEGG WNT Annotation
SIX1	NM_005982.3	-1.73	0.152	0.436	Development & Differentiation, Migration, Transcription Factors
CREBBP	NM_001079846.1	0.226	0.156	0.436	KEGG WNT Annotation
AXIN2	NM_004655.3	1.83	0.157	0.436	Canonical Wnt Pathway, KEGG WNT Annotation, WNT Signaling Negative Regulation, WNT Signaling Target Genes
IGF1	NM_000618.3	1.5	0.159	0.436	Cell Cycle, Development & Differentiation, Migration
PPP3R1	NM_000945.3	0.189	0.16	0.436	KEGG WNT Annotation
TCF7	NM_003202.2	-0.773	0.162	0.436	Canonical Wnt Pathway, KEGG WNT Annotation, Transcription Factors
SMAD3	NM_005902.3	0.671	0.164	0.436	KEGG WNT Annotation
AES	NM_001130.5	0.196	0.165	0.436	Canonical Wnt Pathway
NRP1	NM_003873.5	-3.11	0.166	0.436	Development & Differentiation, Migration
WNT7A	NM_004625.3	1.36	0.167	0.436	Calcium Binding and Signaling, Canonical Wnt Pathway, KEGG WNT Annotation
FN1	NM_212482.1	-1.11	0.17	0.436	Development & Differentiation, Migration
FOSL1	NM_005438.2	-0.633	0.18	0.447	Cell Cycle, KEGG WNT Annotation, Transcription Factors, WNT Signaling Target Genes
SMAD4	NM_005359.3	0.161	0.18	0.447	KEGG WNT Annotation
MYLK	NM_053032.2	0.378	0.182	0.447	EMT Metastasis

CCND2	NM_001759.2	-0.492	0.192	0.466	Cell Cycle, Development & Differentiation, KEGG WNT Annotation, WNT Signaling Target Genes
WNT5A	NM_003392.3	1.74	0.195	0.466	Calcium Binding and Signaling, KEGG WNT Annotation
STAT3	NM_139276.2	0.477	0.198	0.467	EMT Metastasis
FBXW4	NM_022039.3	0.156	0.205	0.477	WNT Signaling Negative Regulation
SOX9	NM_000346.2	-0.901	0.218	0.5	Adhesion, Development & Differentiation, Transcription Factors
PORCN	NM_022825.2	0.199	0.222	0.504	Canonical Wnt Pathway, KEGG WNT Annotation
POU5F1	NM_002701.4	0.432	0.231	0.517	Development & Differentiation, Transcription Factors
TIMP1	NM_003254.2	-0.631	0.251	0.551	EMT Metastasis
MAPK8	NM_002750.2	-0.349	0.252	0.551	KEGG WNT Annotation, Planar Cell Polarity (PCP)
MMP2	NM_004530.2	-0.968	0.267	0.569	Calcium Binding and Signaling, Proteolysis
CCND3	NM_001760.2	-0.38	0.275	0.58	KEGG WNT Annotation
PDGFRA	NM_006206.3	-0.652	0.286	0.594	Development & Differentiation, Migration
LRP5	NM_002335.1	-0.363	0.304	0.616	Canonical Wnt Pathway, KEGG WNT Annotation
PYGO1	NM_015617.1	0.619	0.306	0.616	Canonical Wnt Pathway
WNT4	NM_030761.3	-0.718	0.317	0.624	Calcium Binding and Signaling, Canonical Wnt Pathway, KEGG WNT Annotation
ZEB1	NM_001128128.1	0.212	0.321	0.624	EMT Metastasis
CDH11	NM_001797.2	-1.13	0.323	0.624	EMT Metastasis
NRCAM	NM_005010.4	1.29	0.327	0.624	Adhesion, Development & Differentiation, Migration
GSK3A	NM_019884.2	-0.056	0.331	0.624	Canonical Wnt Pathway
WNT16	NM_057168.1	0.801	0.331	0.624	Calcium Binding and Signaling, Canonical Wnt Pathway, KEGG WNT Annotation
FBXW11	NM_033645.2	0.0985	0.345	0.643	KEGG WNT Annotation, WNT Signaling Negative Regulation
WNT6	NM_006522.3	1	0.377	0.689	Calcium Binding and Signaling, Canonical Wnt Pathway, KEGG WNT Annotation

CD44	NM_001001392.1	0.28	0.378	0.689	Adhesion, Migration
ID2	NM_002166.4	-0.625	0.392	0.698	Cell Cycle, Development & Differentiation
VEGFA	NM_001025366.1	-0.705	0.395	0.698	Development & Differentiation, Migration
MMP9	NM_004994.2	0.608	0.401	0.698	Calcium Binding and Signaling, Development & Differentiation, Migration, Proteolysis
WNT9B	NM_003396.1	0.553	0.403	0.698	KEGG WNT Annotation
FZD6	NM_003506.2	-0.606	0.403	0.698	Canonical Wnt Pathway, KEGG WNT Annotation
MAPK9	NM_139068.2	0.066	0.406	0.698	KEGG WNT Annotation
BTRC	NM_033637.2	-0.113	0.411	0.698	KEGG WNT Annotation, WNT Signaling Negative Regulation, WNT Signaling Target Genes
PRKACB	NM_182948.2	0.427	0.421	0.7	KEGG WNT Annotation
WNT5B	NM_032642.2	-0.428	0.423	0.7	Calcium Binding and Signaling, KEGG WNT Annotation
ERBB2	NM_001005862.1	-0.242	0.429	0.703	EMTMetastasis
LRP6	NM_002336.1	-0.806	0.434	0.703	Canonical Wnt Pathway, KEGG WNT Annotation, WNT Signaling Negative Regulation
RAC3	NM_005052.2	0.152	0.437	0.703	KEGG WNT Annotation
PLAUR	NM_001005376.1	-0.333	0.446	0.705	Proteolysis
CDH1	NM_004360.2	1	0.448	0.705	Adhesion, Development & Differentiation, EMT Metastasis
CXXC4	NM_025212.1	-0.669	0.454	0.705	Canonical Wnt Pathway, KEGG WNT Annotation, WNT Signaling Negative Regulation
PRKX	NM_005044.1	0.198	0.455	0.705	KEGG WNT Annotation
MYC	NM_002467.3	0.253	0.458	0.705	Cell Cycle, KEGG WNT Annotation, Transcription Factors, WNT Signaling Target Genes
APC	NM_000038.3	0.0957	0.477	0.725	Canonical Wnt Pathway, KEGG WNT Annotation, WNT Signaling Negative Regulation
RAC1	NM_198829.1	-0.0704	0.479	0.725	KEGG WNT Annotation

VANGL1	NM_138959.2	-0.341	0.5	0.747	KEGG WNT Annotation
SFRP4	NM_003014.2	0.781	0.507	0.747	Canonical Wnt Pathway, KEGG WNT Annotation, WNT Signaling Negative Regulation
FGF7	NM_002009.3	0.585	0.51	0.747	Migration
RHOA	NM_001664.2	-0.0851	0.518	0.747	KEGG WNT Annotation, Planar Cell Polarity (PCP)
RBX1	NM_014248.2	-0.0823	0.526	0.747	KEGG WNT Annotation
SOST	NM_025237.2	0.585	0.527	0.747	Canonical Wnt Pathway
PPP3CB	NM_001142354.1	0.0756	0.528	0.747	KEGG WNT Annotation
NFATC1	NM_172389.1	-0.301	0.528	0.747	Calcium Binding and Signaling, KEGG WNT Annotation
PKN1	NM_213560.1	0.0702	0.536	0.749	EMTMetastasis
WIF1	NM_007191.2	0.329	0.537	0.749	Canonical Wnt Pathway, KEGG WNT Annotation, WNT Signaling Negative Regulation
EP300	NM_001429.2	0.109	0.555	0.764	Canonical Wnt Pathway, KEGG WNT Annotation
FZD2	NM_001466.2	0.506	0.557	0.764	Calcium Binding and Signaling, Canonical Wnt Pathway, KEGG WNT Annotation
AHR	NM_001621.3	-0.418	0.567	0.772	Cell Cycle, Transcription Factors
WNT7B	NM_058238.1	0.656	0.586	0.79	Calcium Binding and Signaling, Canonical Wnt Pathway, KEGG WNT Annotation
CSNK2A1	NM_177559.2	0.0647	0.589	0.79	Canonical Wnt Pathway, KEGG WNT Annotation
WNT10A	NM_025216.2	-0.278	0.611	0.813	Calcium Binding and Signaling, Canonical Wnt Pathway, KEGG WNT Annotation
PITX2	NM_000325.5	0.467	0.624	0.825	Transcription Factors, WNT Signaling Target Genes
DKK4	NM_014420.2	-0.225	0.641	0.833	KEGG WNT Annotation
SFRP2	NM_003013.2	-0.341	0.646	0.833	KEGG WNT Annotation
CEBPD	NM_005195.3	-0.269	0.646	0.833	Transcription Factors
DVL1	NM_004421.2	0.0857	0.649	0.833	Canonical Wnt Pathway, KEGG WNT Annotation, Planar Cell Polarity (PCP)

ETS2	NM_005239.4	-0.205	0.657	0.837	Transcription Factors
FZD4	NM_012193.2	0.369	0.67	0.848	Canonical Wnt Pathway, KEGG WNT Annotation
PRKCB	NM_212535.1	-0.126	0.684	0.858	KEGG WNT Annotation
FZD1	NM_003505.1	0.292	0.698	0.868	Canonical Wnt Pathway, KEGG WNT Annotation
BAMBI	NM_012342.2	0.492	0.702	0.868	Canonical Wnt Pathway
CCND1	NM_053056.2	-0.339	0.722	0.887	Calcium Binding and Signaling, Cell Cycle, Development & Differentiation, KEGG WNT Annotation, WNT Signaling Negative Regulation, WNT Signaling Target Genes
AXIN1	NM_181050.1	0.0343	0.746	0.899	Canonical Wnt Pathway, KEGG WNT Annotation, WNT Signaling Negative Regulation
WNT3	NM_030753.3	0.189	0.747	0.899	Calcium Binding and Signaling, Canonical Wnt Pathway, KEGG WNT Annotation
GSK3B	NM_002093.2	-0.0331	0.754	0.899	Canonical Wnt Pathway, KEGG WNT Annotation
WNT11	NM_004626.2	-0.381	0.756	0.899	Calcium Binding and Signaling, KEGG WNT Annotation
FZD7	NM_003507.1	-0.288	0.757	0.899	Canonical Wnt Pathway, KEGG WNT Annotation
SMO	NM_005631.3	-0.172	0.772	0.911	Development & Differentiation, Migration
CAMK2B	NM_001220.3	-0.216	0.787	0.923	KEGG WNT Annotation
T	NM_003181.2	0.343	0.802	0.933	Development & Differentiation, Transcription Factors
FRZB	NM_001463.2	0.222	0.808	0.935	WNT Signaling Negative Regulation
TGFB1	NM_000660.3	-0.0305	0.819	0.937	EMT Metastasis
CTNNB1	NM_001098210.1	0.0236	0.827	0.937	Canonical Wnt Pathway, KEGG WNT Annotation
EGR1	NM_001964.2	0.206	0.828	0.937	Development & Differentiation, Transcription Factors
GPC4	NM_001448.2	-0.519	0.837	0.937	Canonical Wnt Pathway
PRKCA	NM_002737.2	0.159	0.837	0.937	KEGG WNT Annotation
KLF5	NM_001730.3	0.113	0.847	0.937	Transcription Factors
IL6	NM_000600.1	-0.157	0.852	0.937	Development & Differentiation, Migration

FZD9	NM_003508.2	-0.115	0.854	0.937	Canonical Wnt Pathway, KEGG WNT Annotation
ZEB2	NM_014795.3	0.0998	0.857	0.937	EMT Metastasis
JAG1	NM_000214.2	-0.106	0.865	0.939	Development & Differentiation, Migration
EFNB1	NM_004429.4	-0.0881	0.877	0.943	Development & Differentiation, Migration
DKK3	NM_001018057.1	0.0555	0.879	0.943	Canonical Wnt Pathway, WNT Signaling Negative Regulation
SOX17	NM_022454.3	0.0902	0.895	0.953	Canonical Wnt Pathway, KEGG WNT Annotation, WNT Signaling Negative Regulation
NANOG	NM_024865.2	-0.0481	0.899	0.953	Development & Differentiation, Transcription Factors
PRKCG	NM_002739.3	-0.0605	0.931	0.972	KEGG WNT Annotation
CSNK1A1	NM_001892.4	0.00903	0.937	0.972	Canonical Wnt Pathway, KEGG WNT Annotation
TP53	NM_000546.2	0.011	0.942	0.972	KEGG WNT Annotation
WNT2	NM_003391.2	0.0512	0.944	0.972	Calcium Binding and Signaling, Canonical Wnt Pathway, KEGG WNT Annotation
SKP1	NM_170679.2	0.00273	0.978	1	KEGG WNT Annotation
BCL9	NM_004326.2	0.00212	0.994	1	Canonical Wnt Pathway
SNAI1	NM_005985.2	6.97e-16	1	1	EMT Metastasis
**Genes below the background level**					
CTGF	NM_001901.2	<20 counts			Development & Differentiation, Migration, Adhesion
PRKACG	NM_002732.2	<20 counts			KEGG WNT Annotation
DPP10	NM_020868.3	<20 counts			Proteolysis
WNT1	NM_005430.2	<20 counts			Calcium Binding and Signaling, Canonical Wnt Pathway, KEGG WNT Annotation
TWIST1	NM_000474.3	<20 counts			Development & Differentiation, Migration
FGF4	NM_002007.2	<20 counts			Development & Differentiation, Adhesion
MMP3	NM_002422.3	<20 counts			EMT Metastasis
IGF2	NM_001127598.1	<20 counts			Imprinted Gene, Development & Differentiation, Cell Cycle

WNT8A	NM_058244.2	<20 counts			Calcium Binding and Signaling, Canonical Wnt Pathway, KEGG WNT Annotation
PPP3R2	NM_147180.2	<20 counts			KEGG WNT Annotation
TWIST2	NM_057179.2	<20 counts			EMT Metastasis
PROM1	NM_006017.1	<20 counts			EMT Metastasis
PLCB4	NM_000933.3	<20 counts			KEGG WNT Annotation
WNT9A	NM_003395.1	<20 counts			Calcium Binding and Signaling, KEGG WNT Annotation, Planar Cell Polarity (PCP)
DKK2	NM_014421.2	<20 counts			KEGG WNT Annotation
DKK1	NM_012242.2	<20 counts			Canonical Wnt Pathway, KEGG WNT Annotation, WNT Signaling Negative Regulation
WISP1	NM_080838.1	<20 counts			WNT Signaling Target Genes
**HOUSEKEEPING IN THE PANEL**					
CC2D1B	NM_032449.2	Housekeeping			
COG7	NM_153603.3	Housekeeping			
EDC3	NM_001142443.1	Housekeeping			
GPATCH3	NM_022078.2	Housekeeping			
HDAC3	NM_003883.2	Housekeeping			
MTMR14	NM_022485.3	Housekeeping			
NUBP1	NM_001278506.1	Housekeeping			
PRPF38A	NM_032864.3	Housekeeping			
SAP130	NM_024545.3	Housekeeping			
SF3A3	NM_006802.2	Housekeeping			
TLK2	NM_006852.2	Housekeeping			
ZC3H14	NM_001160103.1	Housekeeping			

Abbreviations: KEGG, Kyoto Encyclopedia of Genes and Genomes; EMT, Epithelial mesenchymal transition
