# Supplementary material for: Cohesin Mutations Induce Chromatin Conformation Perturbation of the H19/IGF2 Imprinted Region and Gene Expression Dysregulation in Cornelia de Lange Syndrome Cell Lines
Source: Biomolecules. 2021 Nov 2;11(11):1622. doi: 10.3390/biom11111622 (PMC8615450; doi:10.3390/biom11111622)
Supplement: Supplementary file 1 [file biomolecules-11-01622-s001.zip › Table S3.pdf]

Table S3. Quantitative CpGs methylation results of *IGF2/H19* (IC1), *MEST*, *PEG10, GNAS and GNAS-AS1* DMRs in CTRL and CdLS cell lines

**Sample**	**Mean Methylation Levels (%)**				
	**ICR1**	***MEST*-DMR**	***PEG10*-DMR**	***GNAS*-DMR**	***GNAS-AS1*-DMR**
CTRL1	41	42	42	31	22
CTRL2	36	43	43	37	10
CTRL3	34	47	35	38	21
CTRL4	38	45	40	N.A.	N.A.
CdLS1	38	36	41	32	30
CdLS2	9	33	43	37	30
CdLS3	43	60	44	20	23
CdLS4	44	47	44	31	37
CdLS5	44	52	43	31	30
CdLS6	40	40	41	24	33
CdLS7	42	35	42	4	8
CdLS8	45	50	41	26	35
CdLS9	29	37	41	42	30

The results are the mean of two independent pyrosequencing experiments
N.A., not analyzable
